# Development of machine learning models for the screening of potential HSP90 inhibitors

**DOI:** 10.3389/fmolb.2022.967510

**Published:** 2022-10-19

**Authors:** Mohd Imran Khan, Taehwan Park, Mohammad Azhar Imran, Venu Venkatarame Gowda Saralamma, Duk Chul Lee, Jaehyuk Choi, Mohammad Hassan Baig, Jae-June Dong

**Affiliations:** ^1^ Department of Family Medicine, Gangnam Severance Hospital, Yonsei University College of Medicine, Seoul, South Korea; ^2^ Department of Family Medicine, Severance Hospital, Yonsei University College of Medicine, Seoul, South Korea; ^3^ BNJBiopharma, Yonsei University International Campus, Incheon, South Korea

**Keywords:** cancer, machine learing, pharmacophore, Hsp90, virtual screeening

## Abstract

Heat shock protein 90 (Hsp90) is a molecular chaperone playing a significant role in the folding of client proteins. This cellular protein is linked to the progression of several cancer types, including breast cancer, lung cancer, and gastrointestinal stromal tumors. Several oncogenic kinases are Hsp90 clients and their activity depends on this molecular chaperone. This makes HSP90 a prominent therapeutic target for cancer treatment. Studies have confirmed the inhibition of HSP90 as a striking therapeutic treatment for cancer management. In this study, we have utilized machine learning and different *in silico* approaches to screen the KCB database to identify the potential HSP90 inhibitors. Further evaluation of these inhibitors on various cancer cell lines showed favorable inhibitory activity. These inhibitors could serve as a basis for future development of effective HSP90 inhibitors.

## Introduction

Due to their distinct cellular distribution, molecular chaperones have emerged as appealing drug targets for therapeutic research (Schopf et al., 2017; Kim et al., 2018; Li et al., 2018). Heat-shock proteins, a molecular chaperone, vary from 10 kDa to >100 kDa in size and are located across different cellular compartments. HSP90α and HSP90β in the cytoplasm, GRP94 in the endoplasmic reticulum, and TRAP-1 (tumor necrosis factor receptor-associated protein 1) in the mitochondria are different isoforms of 90 kDa HSPs (Hoter et al., 2018).

Hsp90 reportedly involves a wide range of essential cell functions (Wu et al., 2017). It plays a crucial role in the folding and stabilizing of more than 200 client proteins (Trepel et al., 2010; Azoitei et al., 2014). Hsp90 is well reported to be involved in various cancer progression by playing a prominent role in the biological functions and maintaining the conformation of several oncoproteins (Boroumand et al., 2018; Liew et al., 2022). The role of HSP90 has been thoroughly investigated and linked with progression of various cancer types, including breast cancer, lung cancer, melanoma, and gastrointestinal stromal tumors (Whitesell and Lindquist, 2005; Sherman and Multhoff, 2007; Wandinger et al., 2008; Graner, 2016; Chatterjee and Burns, 2017; Wu et al., 2017). Studies have well established that compared to normal cells, the expression level of Hsp90 is much higher in various cancer cells (Beliakoff and Whitesell, 2004; Mahalingam et al., 2009; Moser et al., 2009; Miyata et al., 2013; Sanchez et al., 2020). In cancer cells, the inhibition of Hsp90 promotes the degradation of client oncoproteins, thereby making it a significant cancer therapeutic strategy (Bhat et al., 2014; Ozgur and Tutar, 2016; Liu et al., 2019). In addition, the role of Hsp90 is reported in several other diseases (Cowen et al., 2009; Lackie et al., 2017; Wang et al., 2017). It is widely investigated as a crucial therapeutic drug target for viral, parasitic, fungal, and various neurodegenerative disorders (Ernst et al., 2014; Lackie et al., 2017).

Hsp90 is considered a prominent anticancer drug target because of its interaction with several kinases (almost 60% kinases) (Taipale et al., 2012). The inhibition of HSP90 results in the degradation of the client kinases (mediated by ubiquitination). The central role of HSP90 in these tumor mediating interactions has placed it as one of the most prominent anticancer drug targets. This has also constantly developed research interest towards developing Hsp90 targeting anticancer agents (Bhat et al., 2014; Kumalo et al., 2015). In the early 1990s, researchers identified the druggable pocket of HSP90 (Sanchez et al., 2020). Since then, many inhibitors targeting the N-terminal domain have been developed, some of which are also under clinical trials (Yuno et al., 2018). Despite so much research, there is still no HSP90 targeting molecule approved by FDA for cancer monotherapy.

The recent advancement in technology has fastened the drug discovery process. A large number of molecules have been developed using advanced computational techniques (Baig et al., 2016; Lin et al., 2020). Virtual screening is an efficient and cost-effective strategy for identifying chemical moieties and structural scaffolds potentially crucial for the binding to a target protein (Lionta et al., 2014) (Baig et al., 2016).

We used three different ML techniques on the ChEMBL activity dataset along with DUD-E decoys to increase the performance and balance out the number bias. The relevant features were selected to develop a robust model for HSP90. The best ML model was chosen on several parameters and was further utilized for screening the Korean Chemical Bank (https://chembank.org/) to identify the potential HSP90 inhibitor. Further various *in silico* techniques were applied to evaluate the selected compounds’ binding potential and stability against HSP90. Finally, the top compounds were experimentally validated. The outcome of this study results in identifying potent HSP90 inhibitors with novel scaffolds.

## Material methods

### Dataset

ChEMBL database (Mendez et al., 2019) was used to select the dataset of molecules having previously reported activity against HSP90 (ChEMBL ID: CHEMBL3880). The selected assay was a single protein assay designated to HSP 90-alpha. The dataset was filtered for (i). Organism: human and (ii). Binding activity type: IC50.

Further the data was preprocessed and filtered for desired information. The unique molecules were segregated to remove the redundancy. To make a classification model, molecules having IC50 activity value less than or equal to 100 nM were treated as active, and more than 500 nM were treated as inactive. Decoys were generated by the DUD-E database (Mysinger et al., 2012). Both active and inactive datasets were initially segregated in smiles format.

### Descriptor calculation and feature selection

RDkit was used to calculate the MACCS KEYS, ECFP4, and 1D&2D descriptors. The biophysical molecular descriptors and fingerprints comprising 1385 unique properties were calculated. To reduce the complexity, improve predictive power, and reduce overfitting of the model, feature selection was performed. Finally, a total of 62 relevant features were selected with Pearson correlation coefficient more than 0.3.

### Model building and validation

ML model was built using Python, Scikit-learn (Bac et al., 2021). The dataset was divided into training and test sets in a ratio of 20:80 using the stratified method. Three ML models were built: Randomforest, XGBoost, and SVM (Support Vector Machine). The model data was preserved in the github repository (https://github.com/marinewhlae/HSP90). Hyperparameter tuning was done by the grid search cross-validation method.

The model was validated through several performance parameters, such as Accuracy, Precision, Recall, MCC (Matthews correlation coefficient), F1 score, and ROC-AUC (Receiver Operating Characteristic curve - Area Under the Curve).

$$ACC=\frac{TP+TN}{TP+TN+FP+FN},\;Precision=\frac{TP}{TP+FP},\;Recall=\frac{TP}{TP+FN}$$

$$F_{1}=2\times \frac{PPV\times TPR}{PPV+TPR}=\frac{2TP}{2TP+FP+FN},\;MCC=\frac{TP\times TN-FP\times FN}{\sqrt{\left(TP+FP\right)\left(TP+FN\right)\left(TN+FP\right)\left(TN+FN\right)}}$$

### Y-scrambling

Y-scrambling is one of the methods used to verify machine learning models and is called Y-randomization or Y-permutation. By randomly mixing response variables (Y data) and intentionally breaking the connection between feature variables (X data), this is an attempt to verify the accidental action of coincidence in model performance, i.e., whether the model’s performance on selected dataset was a random accidental event.

Randomly mix the Y value to create a pair mixed with the existing X value for model learning.

This process is repeated 1000 times, and the performance metric (Accuracy, MCC, F1 score) is compared with the existing learned model.

If the performance of a model learned with existing data is similar to or inferior to that of a model learned through y-scrambling, even if the performance metric of the model is high, the prediction is unreliable.

### ML screening

The selected ML model was used for screening the KCB. KCB is a collection of highly active 578,112 structures. The database was also pre-processed to remove the redundancy and the descriptor information required for screening purposes. The ML model was applied to the compound library, and the molecules predicted to be over 90% active were selected.

### Molecular docking

The structure of Human HSP90 in Complex with Geldanamycin was extracted from the RCSB Protein Databank (PDB ID: 1YET) (Stebbins et al., 1997). HSP90 and Geldanamycin were separated and were subjected to redock using CCDC GOLD v5.8.1 (Jones et al., 1997). The binding orientation of the redock and crystal confirmation of Geldanamycin within the binding site of HSP90 were compared. The molecules identified from the ML approach were further screened against HSP90 based on molecular docking study using CCDC GOLD. The molecules with a PLP fitness score higher than Geldanamycin were selected and further evaluated using autodock (Morris et al., 2009).

### Molecular dynamics simulations

The high performing molecules in complex with the target protein were then subjected to molecular dynamic simulation studies. The thermal stability, binding affinity, and relative motion were the prime objective. Gromacs (v 2020.04 package) (Van Der Spoel et al., 2005) was used to perform the MD throughout the study. CHARMM27 force field and TIP3P water model was applied to the cubic simulation box of 1.2 Å radius. Ligand topology was generated at SwissParam. The simulation box was filled with solvent; and subsequently, the system was electro neutralized using sodium and chloride ions. The bad contacts were corrected through a steepest descent minimization algorithm fixing a maximum force and steps at 1000 kJ/mol/nm and 50,000 respectively. Two rounds of 100 ps of equilibration was performed. First, an isothermal and isochoric equilibration (NVT) was done using Particle Mesh Ewald electrostatics, followed by an isothermal and isobaric equilibration (NPT). Temperature coupling was applied to rectify the temperature differences. Finally, the production MD was performed for 100 ns using trajectories generated after NPT equilibration.

### Free energy calculation

The MMPBSA.py module utilizing the AMBER software was used to appraise the Molecular Mechanic/Poisson-Boltzmann Surface Area (MM-PBSA) (Miller et al., 2012). The binding free energy between the ligand and the receptor was calculated by keeping the account for the vacuum potential energy and solvation free energy terms. Poisson–Boltzmann equation and solvent-accessible surface area (SASA) methods were harnessed to estimate polar and nonpolar energies.

This approach calculates the binding free energy (ΔG binding) based on the following equation:

$$∆G_{binding}=∆G_{MM\;\left(Potential\;energy\;in\;vaccum\right)}+∆G_{sol\;\left(solvation\;effects\right)}$$ (1)

where

$$∆G_{MM}=∆G_{coulomb\;\left(electrostatic\;interaction\right)}+∆G_{Vdw}$$ (2)

and

$$∆G_{sol}=∆G_{polar}+∆G_{nonpolar}$$ (3)

where ΔGpolar represents the electrostatic and ΔGnon-polar is the nonpolar contribution to the solvation free energy.

### Reagents and cells

3-(4,5-imethylthiazol-2-yl)-2,5-diphenyltetrazolium bromide (MTT), Dimethyl sulphoxide (DMSO) were purchased from Sigma-Aldrich. RPMI-1640 medium, fetal bovine serum (FBS), penicillin/streptomycin, trypsin–EDTA, and phosphate buffer saline (PBS) were purchased from Gibco Life Technology. All the HSP90 inhibitors (Compound-1, 2, 3, and 4) were provided by the KCB, Republic of Korea. HSP90 inhibitors were prepared in DMSO at 10 mM stock solution and stored at −20°C. Human breast cancer cells MDA-MB-231 and human lung cancer cells A549 were obtained from Korean Cell Line Bank.

### Cell culture

Human breast cancer cells (MDA-MB-231) and lung cancer cells (A549) were grown in RPMI-1640 medium with 10% heat-inactivated fetal bovine serum (FBS) and 100U/ml of penicillin and 10 μg/ml streptomycin at 37°C in a humidified atmosphere with 5% CO_2_ in the incubator.

### 3-(4,5- imethylthiazol-2-yl)-2,5-diphenyltetrazolium bromide assay

Antiproliferative effects of all the compounds on the MDA-MB-231 and A549 cell line were evaluated by 3-(4,5- imethylthiazol-2-yl)-2,5-diphenyltetrazolium bromide (MTT) assay. In brief, MDA-MB-231 and A549 cells were seeded into 96-well plates (10,000 cells/well) and were allowed to adhere overnight. The cells were then treated with the top 4 selected HSP90 inhibitors (Compound-1, 2, 3, and 4) at various concentrations (0.321µM, 0.625µM, 1.25µM, 2.5µM, and 5 µM) for 72 h. To measure the cell viability, 10 µl of MTT (5 mg/ml) solution was added to each well, and the cells were further incubated for another 3 h at 37 °C. The supernatant was removed and subsequently 200 μl of DMSO was added to each well to dissolve the formazan product. Absorbance at 540 nm was measured using a microplate reader (Promega, Discover), and percentage of cell viability was calculated as follows: (optical density of experimental sample/optical density of control) * 100.

## Result and discussion

### Model development and evaluation

The use of Machine learning techniques in drug discovery has attracted much research interest (Sato et al., 2010; Carpenter and Huang, 2018; Zhu et al., 2020). ML improves the decision-making in hit discovery to retrieve accurate outcomes (Adeshina et al., 2020; Dara et al., 2021). This study aimed to build a robust classification model for screening the potential hits for HSP90. At the outset, this study incorporated a total of 354 inhibitors with reported inhibitory activities against HSP90 from the ChEMBL Bioassay database.

**FIGURE 1:**
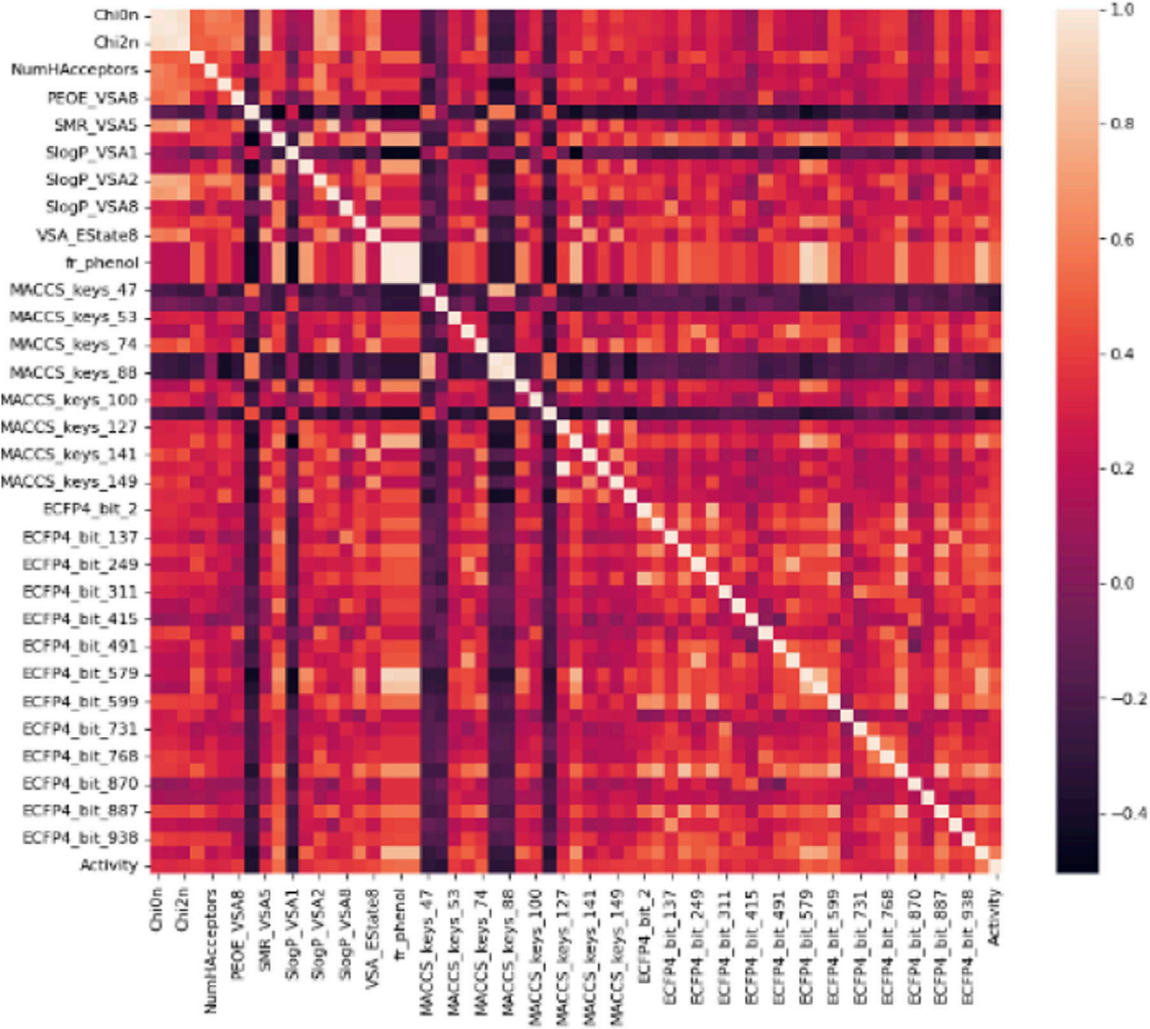
A correlation histogram of selected descriptors against the inhibitory activity. The scale represents the value of the Pearson correlation coefficient.

The primary dataset for molecular descriptors calculation contained 218 active and 136 inactive, and 518 decoy entries (Table 1). The training and test data distribution was in the ratio of 20:80. The magnitude of the biological activity range was sufficiently wide and evenly distributed between the sampling sets. Therefore, the multi-dimensional chemical property space was evenly rendered between training and test sets. In total, 1385 1D&2D, MACCS KEYS, and ECFP4 unique descriptors were calculated (Table 2). Molecular descriptors represent a molecule’s physical, chemical, or topological features that are experimentally or theoretically defined (Todeschini and Consonni, 2008; Dong et al., 2015). The descriptors were filtered using the Pearson correlation coefficient matrix. The relevant features with characteristic correlation with the continuous data of inhibitory activity and low dimensionality were considered as an independent chemical variables. The descriptors that contained a correlation value greater than 0.3 were selected. In this study, 62 high correlating features were used for the classification. A plot for the Pearson correlation coefficient matrix for adopted 1D&2D (20), MACCS KEYS (16) and ECFP4 (26) descriptors against their inhibitory activities are shown in Figure 1. A significant correlation was found between inhibitory activity and selected features.

**TABLE 1:** Training and test dataset used in our study.

	Active	Inactive		Total
	IC_50_<= 100 nM	IC_50_ > 500 nM	Decoy	
No. of molecules	**218**	**136**	**518**	**872**

**TABLE 2:** Molecular descriptor statistics of the dataset.

	Total descriptors	Selected descriptors
1D&2D descriptors	**195**	**20**
MACCS KEYS	**166**	**16**
ECFP4	**1024**	**26**

**FIGURE 2:**
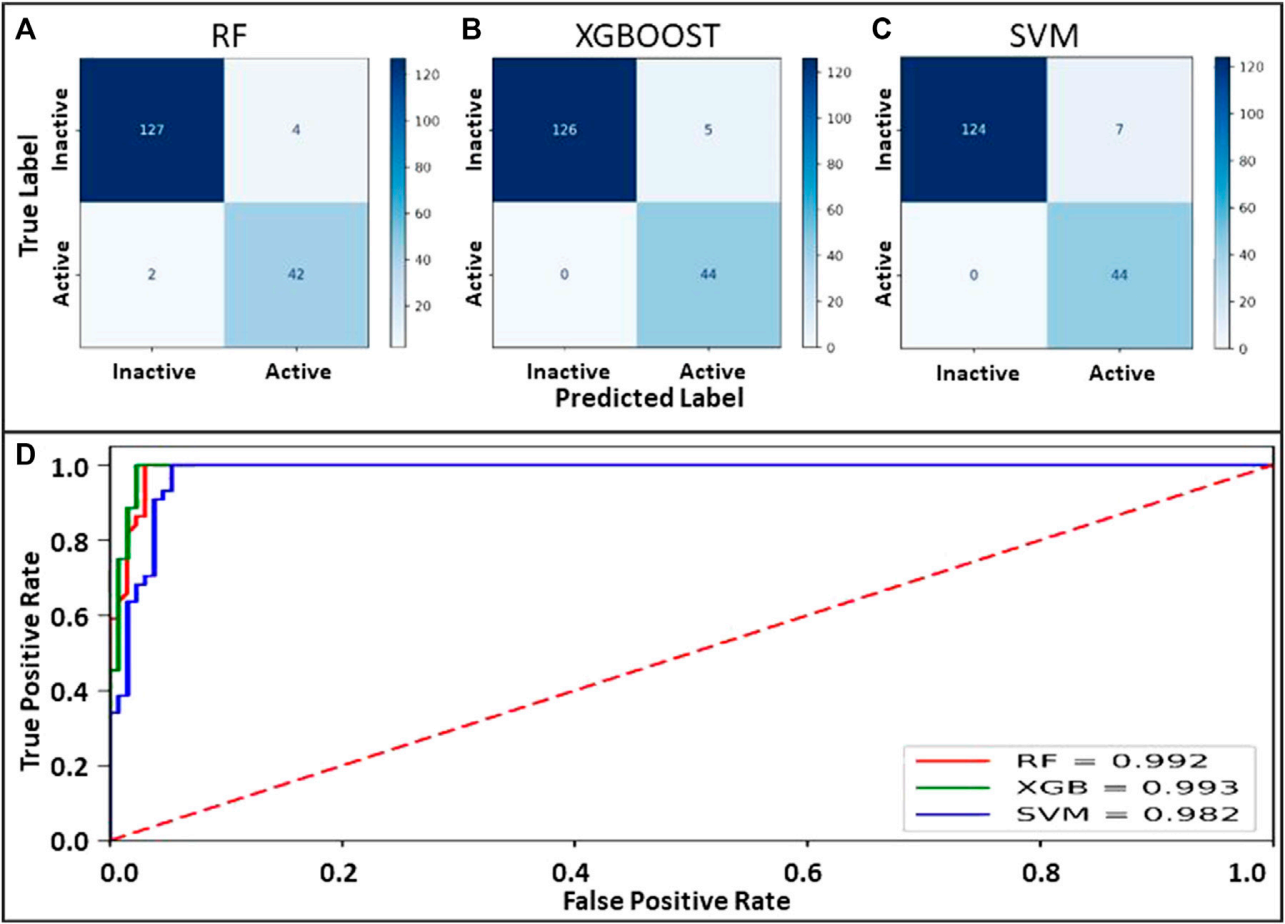
**(A–C)** Confusion matrix for the models prepared using RF, XGBOOST and SVM. **(D)**: ROC curve showing the performance of binary class models.

Machine learning models employing SVM, Random Forest, and XGBoost were generated on the identical binary dataset. The selected parameter sets were optimized to attain maximum accuracy for each classifier. Internal 10-fold cross-validation to tune the hyperparameters was performed. Data was compared to the original class label for all the classifiers to evaluate true positives, true negatives, false positives, and false negatives. The confusion matrix and the evaluated quality parameters of the classifier are illustrated in Figure 2.

The performance of the binary classifier was also analyzed on a comparative ROC plot drawn between the proportion of truly predicted positive classes among all actual positive classes and the proportion of incorrectly predicted negative classes among all actual negatives to encapsulate the findings in confusion matrices.

The accuracy evaluation for the internal ten-fold cross-validated SVM, RF, and XGB was computed while keeping the complexity parameter to 1.0 (Table 3). Scores for six performance parameters viz., accuracy, precision, recall, MCC, F1, and ROC-AUC for the 3 different methods were compared to each other. The ROC-AUC for the XCBoost model was significantly highest among the 3 models. Concretely, the tradeoff between statistical results of the performance parameters among the 3 classifiers establishes an XGBoost model as the best-suited binary class prediction method for our dataset.

**TABLE 3:** The performance parameter collation for the Ramdom forest, XGBoost and Support Vector Machine models.

	Accuracy	Precision	Recall	MCC	F1	ROC-AUC
RF	**0.965**	**0.913**	**0.954**	**0.910**	**0.933**	**0.992**
XGB	**0.971**	**0.898**	**1**	**0.929**	**0.946**	**0.993**
SVM	**0.96**	**0.8622**	**1**	**0.903**	**0.926**	**0.982**

**FIGURE 3:**
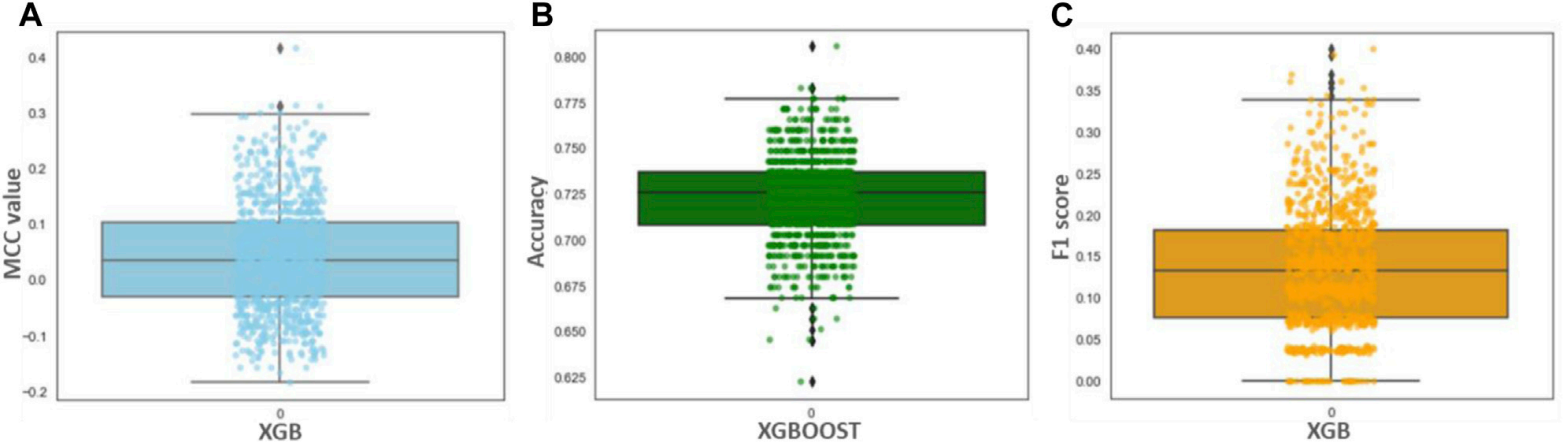
**(A–C)** showing Y-scrambling MCC, Accuracy, and F1 score for the XGBoost model.

Although the accuracy and MCC scores indicated (Table 3) in all three models are above 0.9, demonstrating their ability to generalize outside the initial dataset characteristics, the difference lies in the results of performance for recall values. We found the lowest recall value for RF (0.954), and the highest recall value possessed by XBG and SVM models (1.0). Overall, the higher value of the five performance metric parameters, including accuracy, precision, recall, MCC, and F1, indicates XBGoost as the most accurate classifier in combination with the selected descriptors (Table 3). We performed Y-scrambling randomization test on the XGB model. The results favor the argument for the model not being a change event (Figure 3).

### Screening database

The test dataset with 577,939 molecules was screened on the XGBoost model. A total of 6807 molecules were predicted as active hits against HSP90 (Table 4).

**TABLE 4:** Test dataset for Machine Learning screening.

	Total	Screening	Active	Probability>90%
No. of molecules	**578,112**	**577,939**	**6807**	**237**

The selected active molecules were further screened against HSP90 using CCDC Gold and Autodock. Finally, the top 4 molecules with binding scores from both the docking software higher than Geldanamycin were selected. The PLP fitness score from Gold, binding energy from Autodock, and Classification Probability score from the ML model for the selected compounds are shown in Table 5.

**TABLE 5:** Machine Learning and molecular docking results for the top 4 molecules.

Compound	Classification probability	GOLD docking score	Autodock docking score
	XGboost	(PLP fitness score)	(kcal/mol)
Compound 1	**0.906**	**77.57**	**−10.95**
Compound 2	**0.913**	**76.49**	**−9.48**
Compound 3	**0.951**	**76.16**	**−8.34**
Compound 4	**0.925**	**76.99**	**−10.66**

**FIGURE 4:**
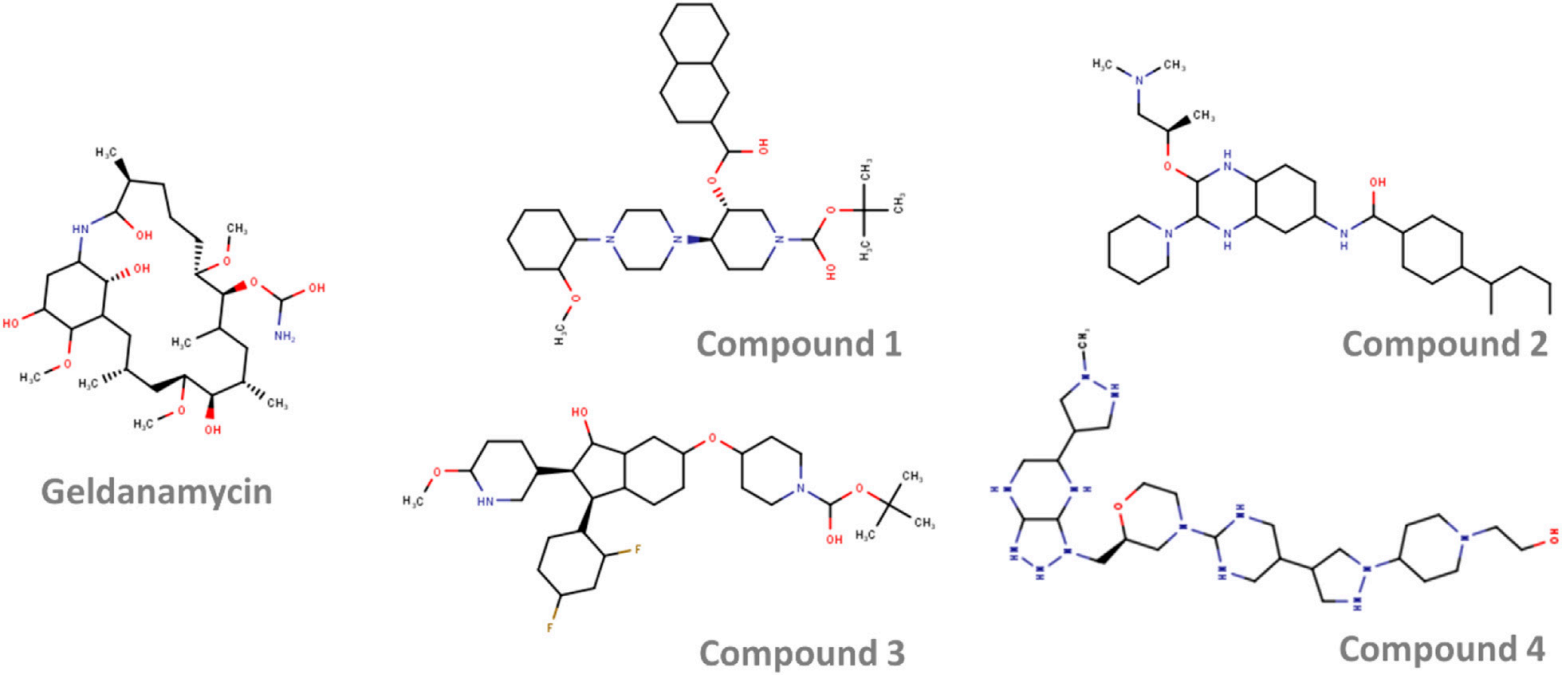
The 2-dimensional structure of the top four selected molecules in screening.

The 2D representation of the four potential molecules selected against HSP90 is shown in Figure 4. When compared with the drug geldanamycin, these molecules showed no significant structural similarity. All the molecules have an extended ring structure with molecular weights ranging from 565.840 Da (Compound 1), 533.846 Da (Compound 2), 572.779 Da (Compound 3), and 593.826 Da (Compound 4).

### Molecular dynamics

**FIGURE 5:**
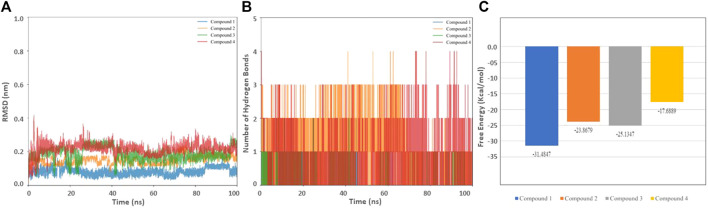
Biophysical simulation analysis of the complexes. **(A)** RMSD plot of HSP90 with the identified compounds **(B)** Hydrogen bond frequency plot **(C)** Binding free energy.

The stability of the selected hits in complex with HSP90 was further confirmed through molecular dynamic simulations (Liu et al., 2018; Naqvi et al., 2018). Apart from stability, we sought to evaluate the binding affinity and the effect of inhibitors on the dynamic structure of the protein. The RMSD, Hydrogen bond, and free energy plot were calculated. (Figure 5; Table 5). The RMSD values for Compounds 1, 2, and 3 were below 2Å, while compound 4 showed a slightly higher fluctuation.

### Principal component analysis

**FIGURE 6:**
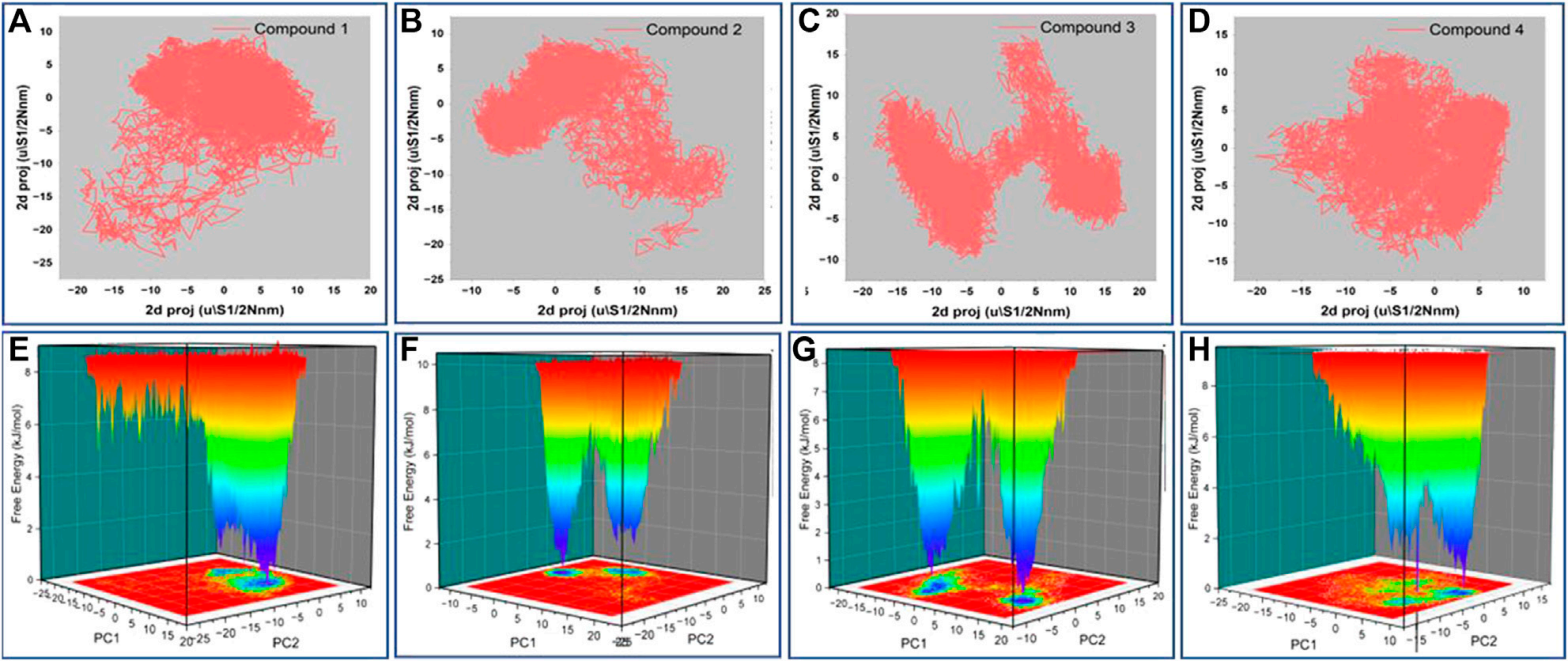
**(A–D)** 2D projection of the trajectory allocation for motion of the proteins in complex with compound 1,2,3 and 4 in the essential subspace, **(E–H)** 3D projection of the free energy landscape as a function of first two principal components.

The principal component analysis (PCA) was carried out on the simulation trajectories to segregate, discover and evaluate the meaningful conformational changes among all the multidirectional atomic thermal fluctuations (David and Jacobs, 2014; Khan et al., 2021). The biggest eigenvectors from the analysis depict the rigorous atomic motion in the complex (Mazanetz et al., 2014). We investigated the projection of 8 eigenvectors for the PCA of HSP90 bound with Gelendamycin and four high-scoring hits (Compound1, 2, 3, and 4). The trajectory suggested different atomic motions during the simulation. The 2D projection of the trajectories in the essential subspace is projected in Figures 6A–D. The results show that the Compound 1 and 2 bound structure of HSP90 occupies a common conformational space, indicating higher complex structural stability.

Further, we plotted the free energy landscapes in Figures 6E–H to understand the folding pattern fluctuations among the top four selected complexes. The simulation ensembles of the four compound bound structure showed narrow peaks with distinct minima among the first two essential components. Complex with compound 1 has most of the ensembles in a narrow range of conformational space with no major state transition. These findings suggest compact packing and better stability of the compound 1 bound structure followed by 2, 3, and 4.

We also determined the stable number of hydrogen bonds stabilizing the interactions between HSP90 and the molecules. As anticipated, Compound 3 and 4 were stabilized with 3–4 stable and consistent hydrogen bonds, indicating their strong binding capability. In drug discovery, the analysis of MM-PBSA is considered an efficient strategy to account binding affinity (Genheden and Ryde, 2015). The binding free energy values for Compounds 1, 2, 3, and 4 were found to be −31.4847, −23.8679, −25.1347, and −17.6889 kcal/mol, respectively (Table 6). The selected compounds were further evaluated on different cancer lines.

**TABLE 6:** Free energy of Binding result of the selected molecules.

MMGBSA	Compound 1	Compound 2	Compound 3	Compound 4
ΔG Binding (Kcal/mol)	−31.4847	−23.8679	−25.1347	−17.6889

### Cellular assay

**FIGURE 7:**
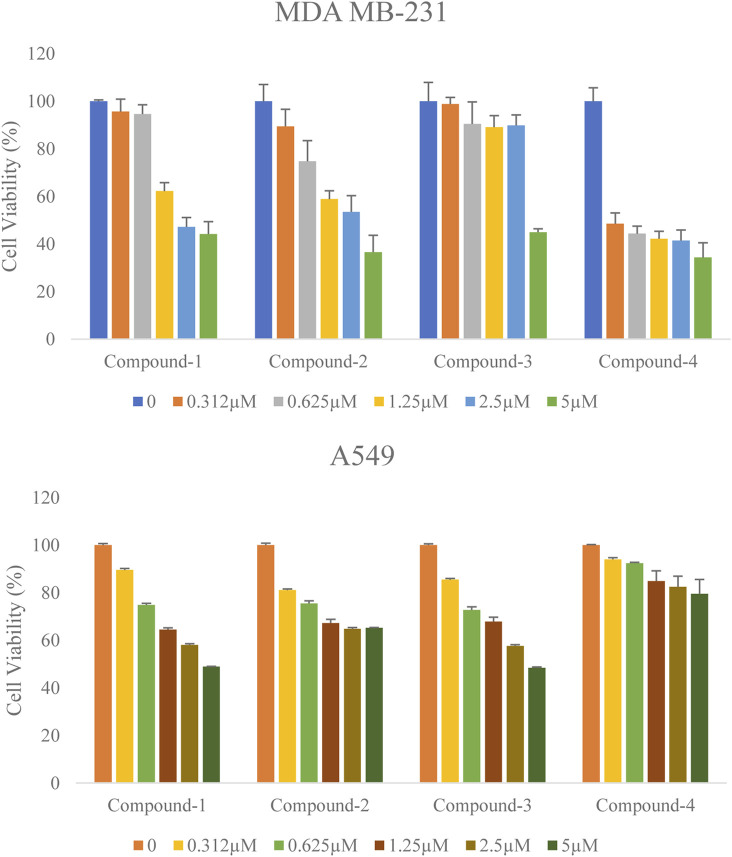
Effect of the selected compounds (Compound-1, 2, 3 and 4) on the cell viability of human breast cancer cells MDA-MB-231 and lung cancer cells A549. Both the cells were treated with various concentrations of these compounds for 72 h. Cell viability was determined by MTT assay. Percentages of viable cells were calculated by comparing treated and solvent control (DMSO) cells. Data are the mean ± S.D. of three replicates.

Antiproliferative activity of the selected compounds was assessed in MDA-MB-231 and A549 cells by MTT assay. The inhibitory effect of these compounds against both cell lines was evaluated using the half-maximal inhibition concentration (IC50) value (a concentration required to inhibit cell growth in 50% of the cell population) at 72 h. Along with the increasing concentration of these compounds (Compound-1, 2, 3, and 4) treatments, all these inhibitors significantly decreased human breast cancer MDA-MB-231 cell and human lung cancer A549 cell proliferation in a dose-dependent manner (Figure 7). The Half inhibitory concentration (IC50) of these compounds (Compound-1, 2, 3, 4) on MDA-MB-231 was 3.62 µM, 3.13 µM, 4.5 µM, and 1.86 µM respectively, and on A549 was 4.13 µM, 6.75 µM, 4.13 µM, and 12.32 µM respectively (Table 7). 17-DMAG is a semi-synthetic geldanamycin analog that binds specifically to Hsp90’s ATP binding site and inhibits the protein folding process. The cell proliferation of MCF-7 and MDA-MB-231 is inhibited by 17-DMAG, with IC50 values of 3.11 μM and 2.16 μM, respectively (Ghadban et al., 2016). Our compounds were found to be demonstrating potency close to 17-DMAG. Overall, the selected compounds (Compound-1, 2, 3, and 4) significantly reduced cell proliferation in MDA-MB-231 and A549 cell lines with low IC50 values at 72 h (Table.7).

**TABLE 7:** IC50 value of HSP90 inhibitors on human cancer cell lines.

Compounds	MDA-MB-231 (IC50 μM)	A549 (IC50 μM)
Compound-1	3.62	4.13
Compound-2	3.13	6.75
Compound-3	4.5	4.13
Compound-4	1.86	12.32

## Conclusion

Heat shock protein 90 (Hsp90) is a molecular chaperone playing a significant role in the folding of client proteins. This cellular protein linkage with cancer progression makes it a viable therapeutic target. This study has utilized machine learning and different in silico approaches to identify potential HSP90 inhibitors. Further experimental validation of the selected compounds on various cancer cell lines proved their anticancer potential. Compound-1 and Compound-3 showed antiproliferative activity with IC50 below 5 µM against both the studied cancer cells. While Compound-2 and 4 also demonstrated good antiproliferative activity against MDA-MB-231 (IC50 < 3.5 µM). The finding of this work identified four novel and promising leads for further development of anticancer drugs targeting Hsp90.

## Data Availability

The original contributions presented in the study are included in the article/Supplementary Material; further inquiries can be directed to the corresponding authors.

## References

[B1] Adeshina Y. O. Deeds E. J. Karanicolas J. (2020). Machine learning classification can reduce false positives in structure-based virtual screening. Proc. Natl. Acad. Sci. U. S. A. 117, 18477–18488. 10.1073/pnas.2000585117 32669436 PMC7414157

[B2] Azoitei N. Diepold K. Brunner C. Rouhi A. Genze F. Becher A. (2014). HSP90 supports tumor growth and angiogenesis through PRKD2 protein stabilization. Cancer Res. 74, 7125–7136. 10.1158/0008-5472.CAN-14-1017 25297628 PMC4315623

[B3] Bac J. Mirkes E. M. Gorban A. N. Tyukin I. Zinovyev A. (2021). Scikit-dimension: A Python package for intrinsic dimension estimation. Entropy (Basel) 23, 1368. 10.3390/e23101368 34682092 PMC8534554

[B4] Baig M. H. Ahmad K. Roy S. Ashraf J. M. Adil M. Siddiqui M. H. (2016). Computer aided drug design: Success and limitations. Curr. Pharm. Des. 22, 572–581. 10.2174/1381612822666151125000550 26601966

[B5] Beliakoff J. Whitesell L. (2004). Hsp90: An emerging target for breast cancer therapy. Anticancer. Drugs 15, 651–662. 10.1097/01.cad.0000136876.11928.be 15269596

[B6] Bhat R. Tummalapalli S. R. Rotella D. P. (2014). Progress in the discovery and development of heat shock protein 90 (Hsp90) inhibitors. J. Med. Chem. 57, 8718–8728. 10.1021/jm500823a 25141341

[B7] Boroumand N. Saghi H. Avan A. Bahreyni A. Ryzhikov M. Khazaei M. (2018). Therapeutic potency of heat-shock protein-90 pharmacological inhibitors in the treatment of gastrointestinal cancer, current status and perspectives. J. Pharm. Pharmacol. 70, 151–158. 10.1111/jphp.12824 28980313

[B8] Carpenter K. A. Huang X. (2018). Machine learning-based virtual screening and its applications to alzheimer's drug discovery: A review. Curr. Pharm. Des. 24, 3347–3358. 10.2174/1381612824666180607124038 29879881 PMC6327115

[B9] Chatterjee S. Burns T. F. (2017). Targeting heat shock proteins in cancer: A promising therapeutic approach. Int. J. Mol. Sci. 18, E1978. 10.3390/ijms18091978 28914774 PMC5618627

[B10] Cowen L. E. Singh S. D. Kohler J. R. Collins C. Zaas A. K. Schell W. A. (2009). Harnessing Hsp90 function as a powerful, broadly effective therapeutic strategy for fungal infectious disease. Proc. Natl. Acad. Sci. U. S. A. 106, 2818–2823. 10.1073/pnas.0813394106 19196973 PMC2650349

[B11] Dara S. Dhamercherla S. Jadav S. S. Babu C. M. Ahsan M. J. (2021). Machine learning in drug discovery: A review. Artif. Intell. Rev. 55, 1947–1999. 10.1007/s10462-021-10058-4 34393317 PMC8356896

[B12] David C. C. Jacobs D. J. (2014). Principal component analysis: A method for determining the essential dynamics of proteins. Methods Mol. Biol. 1084, 193–226. 10.1007/978-1-62703-658-0_11 24061923 PMC4676806

[B13] Dong J. Cao D. S. Miao H. Y. Liu S. Deng B. C. Yun Y. H. (2015). ChemDes: An integrated web-based platform for molecular descriptor and fingerprint computation. J. Cheminform. 7, 60. 10.1186/s13321-015-0109-z 26664458 PMC4674923

[B14] Ernst J. T. Neubert T. Liu M. Sperry S. Zuccola H. Turnbull A. (2014). Identification of novel HSP90α/β isoform selective inhibitors using structure-based drug design. demonstration of potential utility in treating CNS disorders such as Huntington's disease. J. Med. Chem. 57, 3382–3400. 10.1021/jm500042s 24673104

[B15] Genheden S. Ryde U. (2015). The MM/PBSA and MM/GBSA methods to estimate ligand-binding affinities. Expert Opin. Drug Discov. 10, 449–461. 10.1517/17460441.2015.1032936 25835573 PMC4487606

[B16] Ghadban T. Jessen A. Reeh M. Dibbern J. L. Mahner S. Mueller V. (2016). *In vitro* study comparing the efficacy of the water-soluble HSP90 inhibitors, 17-AEPGA and 17-DMAG, with that of the nonwater-soluble HSP90 inhibitor, 17-AAG, in breast cancer cell lines. Int. J. Mol. Med. 38, 1296–1302. 10.3892/ijmm.2016.2696 27498942

[B17] Graner M. W. (2016). HSP90 and immune modulation in cancer. Adv. Cancer Res. 129, 191–224. 10.1016/bs.acr.2015.10.001 26916006

[B18] Hoter A. El-Sabban M. E. Naim H. Y. (2018). The HSP90 family: Structure, regulation, function, and implications in Health and disease. Int. J. Mol. Sci. 19, E2560. 10.3390/ijms19092560 30158430 PMC6164434

[B19] Jones G. Willett P. Glen R. C. Leach A. R. Taylor R. (1997). Development and validation of a genetic algorithm for flexible docking. J. Mol. Biol. 267, 727–748. 10.1006/jmbi.1996.0897 9126849

[B20] Khan M. I. Baig M. H. Mondal T. Alorabi M. Sharma T. Dong J. J. (2021). Impact of the double mutants on spike protein of SARS-CoV-2 B.1.617 lineage on the human ACE2 receptor binding: A structural insight. Viruses 13, 2295. 10.3390/v13112295 34835101 PMC8625741

[B21] Kim H. H. Hyun J. S. Choi J. Choi K. E. Jee J. G. Park S. J. (2018). Structural ensemble-based docking simulation and biophysical studies discovered new inhibitors of Hsp90 N-terminal domain. Sci. Rep. 8, 368. 10.1038/s41598-017-18332-8 29321504 PMC5762686

[B22] Kumalo H. M. Bhakat S. Soliman M. E. (2015). Heat-shock protein 90 (Hsp90) as anticancer target for drug discovery: An ample computational perspective. Chem. Biol. Drug Des. 86, 1131–1160. 10.1111/cbdd.12582 25958815

[B23] Lackie R. E. Maciejewski A. Ostapchenko V. G. Marques-Lopes J. Choy W. Y. Duennwald M. L. (2017). The hsp70/hsp90 chaperone machinery in neurodegenerative diseases. Front. Neurosci. 11, 254. 10.3389/fnins.2017.00254 28559789 PMC5433227

[B25] Li T. Jiang H. L. Tong Y. G. Lu J. J. (2018). Targeting the Hsp90-Cdc37-client protein interaction to disrupt Hsp90 chaperone machinery. J. Hematol. Oncol. 11, 59. 10.1186/s13045-018-0602-8 29699578 PMC5921262

[B26] Liew H. Y. Tan X. Y. Chan H. H. Khaw K. Y. Ong Y. S. (2022). Natural HSP90 inhibitors as a potential therapeutic intervention in treating cancers: A comprehensive review. Pharmacol. Res. 181, 106260. 10.1016/j.phrs.2022.106260 35577308

[B27] Lin X. Li X. Lin X. (2020). A review on applications of computational methods in drug screening and design. Molecules 25, E1375. 10.3390/molecules25061375 32197324 PMC7144386

[B28] Lionta E. Spyrou G. Vassilatis D. K. Cournia Z. (2014). Structure-based virtual screening for drug discovery: Principles, applications and recent advances. Curr. Top. Med. Chem. 14, 1923–1938. 10.2174/1568026614666140929124445 25262799 PMC4443793

[B29] Liu X. Shi D. Zhou S. Liu H. Liu H. Yao X. (2018). Molecular dynamics simulations and novel drug discovery. Expert Opin. Drug Discov. 13, 23–37. 10.1080/17460441.2018.1403419 29139324

[B30] Liu Y. Liu X. Li L. Dai R. Shi M. Xue H. (2019). Identification and structure-activity studies of 1, 3-Dibenzyl-2-aryl imidazolidines as novel Hsp90 inhibitors. Molecules 24, E2105. 10.3390/molecules24112105 31163701 PMC6600241

[B31] Mahalingam D. Swords R. Carew J. S. Nawrocki S. T. Bhalla K. Giles F. J. (2009). Targeting HSP90 for cancer therapy. Br. J. Cancer 100, 1523–1529. 10.1038/sj.bjc.6605066 19401686 PMC2696754

[B54] Mazanetz M. P. Laughton C. A. Fischer P. M. (2014). Investigation of the flexibility of protein kinases implicated in the pathology of Alzheimer’s disease. Molecules. 19 (7), 9134–9159. 10.3390/molecules19079134 24983862 PMC6270768

[B32] Mendez D. Gaulton A. Bento A. P. Chambers J. De Veij M. Felix E. (2019). ChEMBL: Towards direct deposition of bioassay data. Nucleic Acids Res. 47, D930–D940. 10.1093/nar/gky1075 30398643 PMC6323927

[B33] Miller B. R. 3rd McGee T. D. Jr. Swails J. M. Homeyer N. Gohlke H. Roitberg A. E. (2012). MMPBSA.py: An efficient program for end-state free energy calculations. J. Chem. Theory Comput. 8, 3314–3321. 10.1021/ct300418h 26605738

[B34] Miyata Y. Nakamoto H. Neckers L. (2013). The therapeutic target Hsp90 and cancer hallmarks. Curr. Pharm. Des. 19, 347–365. 10.2174/138161213804143725 22920906 PMC7553218

[B55] Morris G. M. Huey R. Lindstrom W. Sanner M. F. Belew R. K. Goodsell D. S. (2009). AutoDock4 and AutoDockTools4: Automated docking with selective receptor flexibility. J. Comput. Chem. 30 (16), 2785–2791. 10.1002/jcc.21256 19399780 PMC2760638

[B35] Moser C. Lang S. A. Stoeltzing O. (2009). Heat-shock protein 90 (Hsp90) as a molecular target for therapy of gastrointestinal cancer. Anticancer Res. 29, 2031–2042. 19528462

[B36] Mysinger M. M. Carchia M. Irwin J. J. Shoichet B. K. (2012). Directory of useful decoys, enhanced (DUD-E): Better ligands and decoys for better benchmarking. J. Med. Chem. 55, 6582–6594. 10.1021/jm300687e 22716043 PMC3405771

[B37] Naqvi A. A. T. Mohammad T. Hasan G. M. Hassan M. I. (2018). Advancements in docking and molecular dynamics simulations towards ligand-receptor interactions and structure-function relationships. Curr. Top. Med. Chem. 18, 1755–1768. 10.2174/1568026618666181025114157 30360721

[B38] Ozgur A. Tutar Y. (2016). Heat shock protein 90 inhibition in cancer drug discovery: From chemistry to futural clinical applications. Anticancer. Agents Med. Chem. 16, 280–290. 10.2174/1871520615666150821093747 26295332

[B39] Sanchez J. Carter T. R. Cohen M. S. Blagg B. S. J. (2020). Old and new approaches to target the Hsp90 chaperone. Curr. Cancer Drug Targets 20, 253–270. 10.2174/1568009619666191202101330 31793427 PMC7502213

[B40] Sato T. Honma T. Yokoyama S. (2010). Combining machine learning and pharmacophore-based interaction fingerprint for *in silico* screening. J. Chem. Inf. Model. 50, 170–185. 10.1021/ci900382e 20038188

[B41] Schopf F. H. Biebl M. M. Buchner J. (2017). The HSP90 chaperone machinery. Nat. Rev. Mol. Cell Biol. 18, 345–360. 10.1038/nrm.2017.20 28429788

[B42] Sherman M. Multhoff G. (2007). Heat shock proteins in cancer. Ann. N. Y. Acad. Sci. 1113, 192–201. 10.1196/annals.1391.030 17978282

[B43] Stebbins C. E. Russo A. A. Schneider C. Rosen N. Hartl F. U. Pavletich N. P. (1997). Crystal structure of an hsp90-geldanamycin complex: Targeting of a protein chaperone by an antitumor agent. Cell 89, 239–250. 10.1016/s0092-8674(00)80203-2 9108479

[B44] Taipale M. Krykbaeva I. Koeva M. Kayatekin C. Westover K. D. Karras G. I. (2012). Quantitative analysis of HSP90-client interactions reveals principles of substrate recognition. Cell 150, 987–1001. 10.1016/j.cell.2012.06.047 22939624 PMC3894786

[B45] Todeschini R. Consonni V. (2008). Handbook of molecular descriptors. Hoboken, New Jersey, US: John Wiley & Sons.

[B46] Trepel J. Mollapour M. Giaccone G. Neckers L. (2010). Targeting the dynamic HSP90 complex in cancer. Nat. Rev. Cancer 10, 537–549. 10.1038/nrc2887 20651736 PMC6778733

[B47] Van Der Spoel D. Lindahl E. Hess B. Groenhof G. Mark A. E. Berendsen H. J. (2005). Gromacs: Fast, flexible, and free. J. Comput. Chem. 26, 1701–1718. 10.1002/jcc.20291 16211538

[B48] Wandinger S. K. Richter K. Buchner J. (2008). The Hsp90 chaperone machinery. J. Biol. Chem. 283, 18473–18477. 10.1074/jbc.R800007200 18442971

[B49] Wang Y. Jin F. Wang R. Li F. Wu Y. Kitazato K. (2017). HSP90: A promising broad-spectrum antiviral drug target. Arch. Virol. 162, 3269–3282. 10.1007/s00705-017-3511-1 28780632

[B50] Whitesell L. Lindquist S. L. (2005). HSP90 and the chaperoning of cancer. Nat. Rev. Cancer 5, 761–772. 10.1038/nrc1716 16175177

[B51] Wu J. Liu T. Rios Z. Mei Q. Lin X. Cao S. (2017). Heat shock proteins and cancer. Trends Pharmacol. Sci. 38, 226–256. 10.1016/j.tips.2016.11.009 28012700

[B52] Yuno A. Lee M. J. Lee S. Tomita Y. Rekhtman D. Moore B. (2018). Clinical evaluation and biomarker profiling of Hsp90 inhibitors. Methods Mol. Biol. 1709, 423–441. 10.1007/978-1-4939-7477-1_29 29177675

[B53] Zhu J. Wu Y. Wang M. Li K. Xu L. Chen Y. (2020). Integrating machine learning-based virtual screening with multiple protein structures and bio-assay evaluation for discovery of novel GSK3β inhibitors. Front. Pharmacol. 11, 566058. 10.3389/fphar.2020.566058 33041806 PMC7517831

